# Segurança e Eficácia da Reabilitação Cardíaca Baseada em Exercício em Pacientes com Angina Refratária

**DOI:** 10.36660/abc.20250331

**Published:** 2026-01-09

**Authors:** Luciana Oliveira Cascaes Dourado, Camila Paixão Jordão, Marcelo Luiz Campos Vieira, Luis Henrique Wolff Gowdak, Carlos Eduardo Negrão, Luiz Antonio Machado Cesar, Luciana Diniz Nagem Janot de Matos

**Affiliations:** 1 Hospital das Clínicas Faculdade de Medicina Universidade de São Paulo São Paulo SP Brasil Instituto do Coração do Hospital das Clínicas da Faculdade de Medicina da Universidade de São Paulo, São Paulo, SP – Brasil; 2 Hospital Israelita Albert Einstein São Paulo SP Brasil Hospital Israelita Albert Einstein, São Paulo, SP – Brasil

**Keywords:** Angina, Reabilitação Cardíaca, Isquemia Miocárdica

## Abstract

**Fundamento:**

As evidências sobre a segurança e os efeitos anti-isquêmicos da reabilitação cardíaca baseada em exercício (RCE) em pacientes com angina refratária (AR) ainda são limitadas.

**Objetivo:**

Avaliar a segurança e a eficácia de um programa de RCE de 12 semanas em pacientes com AR, com foco na melhora dos sintomas, da capacidade funcional e da carga isquêmica avaliada por ecocardiografia de estresse com exercício (EEE).

**Métodos:**

Estudo prospectivo, randomizado, controlado, e unicêntrico, que avaliou um programa de RCE de 12 semanas em pacientes com AR. Quarenta e cinco pacientes foram randomizados para o grupo de reabilitação (GR), que recebeu RCE, ou para o grupo controle (GC), que recebeu apenas tratamento médico. Os desfechos avaliados incluíram mortalidade, eventos cardiovasculares, sintomas anginosos e parâmetros da EEE e do teste cardiopulmonar de exercício (TCPE). O nível de significância estatística adotado foi p < 0,05.

**Resultados:**

Na EEE, a duração do exercício foi significativamente maior no GRpós (após a RCE) em comparação com GRpré (antes da RCE) (∆ = 63,24 ± 19,87 s; p < 0,01). A quantificação de angina foi menor no GRpós em comparação ao GRpré, ao GCpós (após tratamento médico isolado) e ao GCpré (antes do tratamento médico isolado) (∆ = –1,64 ± 0,48 n, p < 0,01; -3,10 ± 0,97 n, p < 0,01; e -2,73 ± 0,92 n, p = 0,01, respectivamente). O limiar de angina foi maior em GRpós em comparação ao GRpré e ao GRpós (∆ = 89,66 ± 33,16 s, p = 0,04; e 111,76 ± 42,25 s, p = 0,04, respectivamente). A melhora na carga isquêmica na EEE foi demonstrada pelo aumento do tempo até o limiar isquêmico GRpós em comparação ao GCpré, e ao GCpré e GRpós (∆ = 83,23 ± 21,84 s, p < 0,01; 98,44 ± 35,11 s, p = 0,03; e 109,34 ± 34,00 s, p < 0,01, respectivamente). No TCPE, o GRpós apresentou aumento na duração do exercício (∆ = 104,54 ± 28,09 s; p < 0,01) e na distância percorrida (∆ = 131,23 ± 30,48 m; p < 0,01) em comparação ao GRpré. Não foram observadas diferenças significativas no VO_2_ entre os grupos. Dois pacientes do grupo CG faleceram. Um paciente do GR apresentou angina prolongada durante o treinamento. Não houve diferenças significativas nos eventos cardiovasculares maiores entre os grupos.

**Conclusão:**

O programa de RCE de 12 semanas foi seguro e eficaz na melhora da duração do exercício, da distância percorrida e da carga isquêmica na EEE em pacientes com AR.

## Graphical abstract

**Figura Central:**
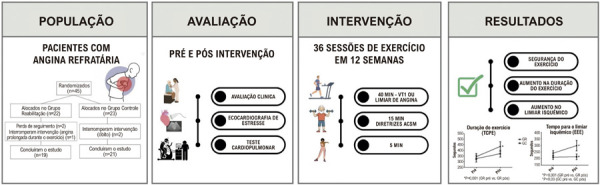
Segurança e Eficácia da Reabilitação Cardíaca Baseada em Exercício em Pacientes com Angina Refratária

## Introdução

A angina refratária (AR) é uma condição crônica e debilitante com duração superior a três meses, caracterizada por angina limitante devido à isquemia miocárdica que não é controlada por meio da combinação de tratamento medicamentoso, angioplastia e cirurgia.^1 , 2^ Pacientes com AR apresentam comprometimento significativo na qualidade de vida,^3^ e diversas terapias alternativas promissoras – algumas ainda experimentais – têm sido testadas com resultados variados.^4 , 5^

Portanto, é urgente a necessidade de incorporar terapias seguras, eficazes e acessíveis para aliviar os sintomas da AR. A reabilitação cardíaca baseada em exercícios (RCE) é considerada o padrão de cuidado na prevenção e tratamento da doença arterial coronariana (DAC) crônica.^6 - 8^ Ela promove adaptações cardiovasculares benéficas que melhoram a aptidão cardiorrespiratória,^9^ exercem efeitos anti-isquêmicos,^10 , 11^ e reduzem eventos cardiovasculares e mortalidade.^12^

Apesar de seus benefícios, a RCE não é frequentemente prescrita a pacientes com AR na prática clínica, devido a preocupações com eventos adversos durante a atividade física.^13^ Tal fato decorre da escassez de evidências que sustentem sua segurança e eficácia no contexto da AR,^14 , 15^ já que esses pacientes geralmente apresentam um limiar isquêmico (LI) baixo e limitações significativas no desempenho físico.^6 , 8 , 13 , 16^

Estudos anteriores demonstraram que o treinamento físico moderado em pacientes com AR pode melhorar a capacidade funcional – medida pelo desempenho no teste de caminhada progressiva ( *Progressive Shuttle Walk* ) – e reduzir a percepção de ameaça da angina, embora não pareça melhorar a percepção de qualidade de vida.^14^ Ainda assim, pouco se sabe sobre a segurança e os efeitos anti-isquêmicos da RCE nessa população, especialmente no que diz respeito à melhora dos sintomas anginosos, da capacidade funcional e da carga isquêmica avaliada por ecocardiografia de estresse com exercício (EEE), quando os pacientes são tratados com terapia médica contemporânea e estratégias de revascularização clinicamente apropriadas.

Neste estudo, testamos a hipótese de que um programa de RCE com duração de 12 semanas é uma estratégia segura e eficaz para melhorar os sintomas anginosos, a capacidade funcional e a carga isquêmica avaliada por EEE em pacientes com AR.

## Métodos

### Desenho do Estudo

Este foi um ensaio clínico randomizado, prospectivo, unicêntrico, que avaliou um programa de RCE de 12 semanas em pacientes com AR. O estudo foi realizado em um hospital universitário público terciário em São Paulo, Brasil (ver Figura Central ). Foi aprovado pelo comitê local de ética e pesquisa (CAAE: 24308213.7.0000.0068) e registrado no ClinicalTrials.gov (NCT03218891). O estudo seguiu os princípios estabelecidos na Declaração de Helsinque. Todos os participantes forneceram consentimento informado por escrito.

### Pacientes do estudo

Foram incluídos pacientes com diagnóstico de AR durante o acompanhamento clínico em um ambulatório especializado. Os participantes elegíveis eram de ambos os sexos, com idade entre 45 e 75 anos, apresentando angina (classe funcional II a IV da *Canadian Cardiovascular Society* [CCS]) persistente por mais de três meses, apesar da terapia médica otimizada (≥3 medicamentos antianginosos). Os pacientes incluídos apresentavam isquemia miocárdica documentada por EEE e não eram elegíveis a procedimentos de revascularização miocárdica cirúrgica ou percutânea.

### Critérios de exclusão

Os critérios de exclusão foram: 1) presença de marca-passo definitivo ou cardiodesfibrilador implantável; 2) ritmo cardíaco não sinusal; 3) histórico de síndrome coronariana aguda ou revascularização miocárdica (cirúrgica ou percutânea) nos últimos três meses; 4) comprometimento funcional causado por qualquer condição clínica que impeça a realização de exercícios; 5) restrição de atividade classificada como Classe D, segundo os critérios da *American Heart Association* para estratificação de risco de eventos durante o exercício.^17 , 18^

### Randomização e intervenção

Os pacientes que atenderam aos critérios de inclusão foram randomizados em blocos para garantir a homogeneidade da amostra, considerando o tamanho da amostra. Uma sequência gerada por computador foi utilizada para alocá-los em um dos dois grupos: 1) grupo de reabilitação (GR): Tratamento clínico otimizado combinado com um programa de treinamento físico de 12 semanas; 2) grupo controle (GC): Tratamento médico otimizado apenas. Todos os pacientes foram submetidos a avaliações clínicas e laboratoriais, teste cardiopulmonar de exercício (TCPE) e EEE tanto na inclusão quanto ao final do estudo.

### Desfechos do estudo

Os desfechos pré-especificados incluíram a avaliação da segurança de um programa de RCE de 12 semanas em pacientes com AR. Essa avaliação focou em eventos clínicos (morte e eventos cardiovasculares maiores), bem como no impacto sobre os sintomas anginosos – classe funcional da angina, número de crises de angina por semana (AAW, do inglês *angina attacks per week* ) e consumo semanal de nitrato de ação rápida (SANCW, do inglês *short-acting nitrate consumption per week* ). Desfechos adicionais incluíram a carga isquêmica miocárdica e a capacidade funcional avaliada por meio de TCPE e EEE.

### Avaliação clínica

Os pacientes foram avaliados em consultas médicas antes e após a intervenção por um cardiologista cego quanto à alocação dos grupos. A classe funcional da angina segundo a CCS foi avaliada. O AAW e o SANC foram registrados em um diário de angina. A adesão à terapia médica foi incentivada durante as consultas, com base na tolerância de cada paciente. A prescrição de medicamentos antianginosos seguiu as diretrizes clínicas vigentes.^7^

### Teste cardiopulmonar de exercício (TCPE)

Para verificar a eficácia e prescrever o treinamento aeróbico (TA), foi realizado o TCPE antes e após a intervenção, utilizando uma esteira motorizada (modelo T2100, GE Healthcare, EUA) e um ergoespirômetro (SensorMedics – VmaxAnalyzer Assembly, Encore 29S, EUA), seguindo um protocolo de exercício incremental (Balke modificado a 2,5 mph). O TCPE foi realizado conforme as diretrizes da *American Heart Association* .^19^ O teste de esforço foi conduzido, interpretado e interrompido de acordo com as Diretrizes Brasileiras para Teste de Esforço.^20^ A capacidade funcional no TCPE foi avaliada por meio de parâmetros de aptidão cardiopulmonar, bem como pelo limiar de angina (LA) durante o exercício e sua quantificação. O LA foi determinado pelo tempo exato (em segundos) e pela frequência cardíaca (FC) no momento em que o paciente relatou sintomas de angina.

### Ecocardiografia de estresse com exercício (EEE)

Para determinar a carga isquêmica, foi realizada uma avaliação ecocardiográfica bidimensional com o aparelho Vivid9 (versão 110.x.x, GE Healthcare), conforme as diretrizes da Sociedade Americana de Ecocardiografia.^21^ A carga isquêmica miocárdica na EEE foi definida por: 1) escore isquêmico, calculado pela diferença entre os escores em esforço e em repouso; e/ou 2) limiar isquêmico (LI), determinado durante a EEE. O teste de esforço foi realizado em um cicloergômetro para membros inferiores, com aumento progressivo da carga de trabalho de 5 para 25 J/s a cada 3 minutos. O teste foi interrompido conforme os critérios das diretrizes.^21 , 22^ Para avaliar a contratilidade segmentar na EEE, foi utilizado um modelo de 17 segmentos, e o índice de escore de movimentação da parede foi definido com base nas diretrizes.^21^ O início da isquemia detectada pela EEE foi determinado pelo momento e pela FC em que começaram as alterações regionais na movimentação da parede miocárdica, sendo considerado o LI. A capacidade funcional na EEE foi avaliada pela duração do exercício, carga máxima atingida, LA (FC e tempo) e quantificação da angina. A angina foi graduada por uma escala subjetiva de dor de 0 (sem dor) a 10 (dor muito intensa).^23^

### Programa de reabilitação cardíaca baseado em exercício (RCE)

O programa de RCE foi realizado em um centro de reabilitação cardiovascular de um hospital terciário, em uma sala de treinamento com temperatura controlada, equipada com bicicletas ergométricas, esteiras e equipamentos para exercícios de força, sob supervisão de profissionais qualificados em reabilitação. O protocolo consistiu em 36 sessões de exercício ao longo de 12 semanas, com frequência de três vezes por semana. Cada sessão supervisionada teve duração de 60 minutos, incluindo: 40 minutos de TA, 15 minutos de treinamento resistido e 5 minutos de alongamento. O componente TA incluiu cinco minutos de aquecimento, 30 minutos de exercício aeróbico contínuo em esteira motorizada e cinco minutos de desaquecimento. A prescrição do TA foi orientada por parâmetros fisiológicos específicos do exercício identificados durante o TCPE,^24^ visando uma FC correspondente, no mínimo, ao primeiro limiar ventilatório (VT1) e/ou ao LA, nos casos em que a FC do LA era inferior à FC do VT1.

O exercício contínuo foi incentivado; no entanto, caso o paciente apresentasse angina leve a moderada (classificada até 3 em uma escala de dor de 0 [sem dor] a 10 [dor intensa]), eram recomendadas breves interrupções ou redução da intensidade.^23^ O exercício era retomado assim que os sintomas cessavam. Os pacientes foram monitorados continuamente por telemetria, e a FC foi registrada no meio e ao final de cada sessão aeróbica para o cálculo da FC média de treinamento. Dinitrato de isossorbida sublingual (5 mg) foi administrado conforme necessário, com base na tolerância individual à angina. Nesses casos, os pacientes eram orientados a não retomar o exercício. As sessões de exercício resistido seguiram as diretrizes mais recentes.^6 , 25^ A intensidade moderada foi adotada com base na escala de percepção de esforço (níveis 4 a 6) e consistiu em duas séries de 8 a 15 repetições. Os exercícios foram realizados de forma rítmica, evitando a apneia e o esforço excessivo (manobra de Valsalva), com expiração durante a fase de contração ou esforço e inspiração durante a fase de relaxamento.^25^

### Análise estatística e cálculo do tamanho amostral

Os dados foram analisados utilizando o software SPSS Statistics 20. As variáveis contínuas foram expressas como média ± desvio padrão (DP) e as variáveis categóricas como porcentagens. As variáveis contínuas que não apresentaram distribuição normal foram descritas por mediana e intervalo interquartil. O teste de Kolmogorov-Smirnov foi utilizado para avaliar a distribuição dos dados. Para comparação entre os grupos, foi aplicado o teste t de Student não pareado ou o teste de Mann-Whitney, conforme a distribuição das variáveis contínuas, e o teste do qui-quadrado para variáveis categóricas, conforme apropriado. Equações de estimação generalizadas (GEE) foram utilizadas para análise de dados longitudinais, e testes post hoc de Bonferroni foram realizados quando diferenças significativas foram identificadas. O nível de significância estatística foi estabelecido em p < 0,05.

Para determinar o tamanho da amostra, baseamo-nos em um estudo anterior^14^ que avaliou a capacidade funcional utilizando o Shuttle Walking Test, assumindo um desvio padrão de 1,2 e uma diferença média entre os grupos de 0,96. Com esses parâmetros, calculamos que seriam necessários 26 participantes em cada grupo (intervenção e controle) para alcançar um poder estatístico de 80% com nível de significância de 5%. O cálculo do tamanho amostral foi realizado utilizando o software Power and Sample Size Calculator (PS), versão 3.0.43.

O tamanho do efeito para os desfechos contínuos foi calculado como a diferença entre as médias marginais estimadas dividida pelo desvio padrão baseado no modelo, uma métrica análoga ao d de Cohen. Essa abordagem considera a correlação intra-indivíduo nos dados longitudinais.

## Resultados

**Figura 1:**
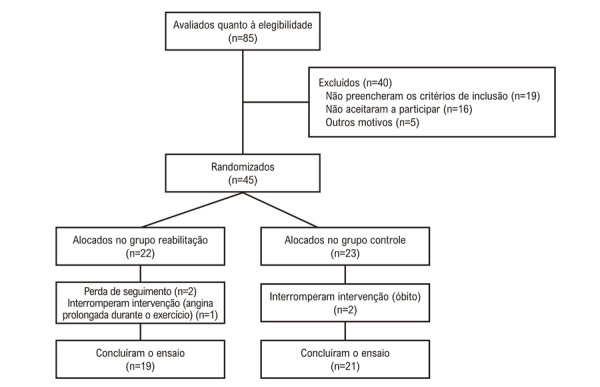
– Fluxograma do grupo reabilitação versus grupo controle.

O fluxograma ( Figura 1 ) ilustra a alocação nos grupos, as perdas e o acompanhamento. Um total de 45 pacientes foi incluído no estudo – 22 alocados para o GR e 23 para o GC.

### Avaliação de segurança

Dois pacientes do GC foram a óbito durante o estudo, sendo um por causa cardiovascular. Um paciente do GR apresentou angina prolongada durante uma sessão de treinamento, foi encaminhado ao departamento de emergência para observação e posteriormente excluído do estudo. Não foram observadas diferenças significativas nos eventos cardiovasculares maiores entre os grupos ao final do estudo ( Tabela 1 ).

**Tabela 1:** – Eventos cardiovasculares durante o período do estudo

Eventos	GR (n=22)	CG (n=23)	Valor p
Óbito, n (%)	0	2 (8,7)	0,5
Morte cardiovascular, n (%)	0	1 (4,3)	> 0,9
Eventos cardiovasculares maiores, n (%)	1 (4,5)	0	0,5

GR: grupo de reabilitação; GC: grupo controle.

### Características basais

Não houve diferenças clínicas significativas entre os grupos no início do estudo ( Tabela 2 ) em relação à classe funcional da angina segundo a classificação da CCS, AAW, SANCW e outros parâmetros clínicos. Ambos os grupos apresentaram fração de ejeção do ventrículo esquerdo (FEVE) média dentro da normalidade. Os pacientes do GR haviam sido submetidos a mais intervenções coronarianas do que os do GC.

**Tabela 2:** – Características clínicas e demográficas basais dos pacientes

Dados basais do paciente	GR (n=22)	GC (n=23)	Valor p
Idade, anos (média ± DP)	61,4±7,9	60,7±9,2	0,8
Sexo masculino (%)	68,2	60,9	0,6
**Achados clínicos**			
Classe funcional da angina CCS (%)			0,7
II	31,8	43,5	
III	27,3	21,7	
IV	40,9	34,8	
AAW, n (mediana, IIQ)	7 (1 - 35)	4 (0,5 - 49)	0,1
SANCW, n (mediana, IIQ)	1,25 (0 – 24,5)	1 (0 – 35)	0,4
SBP, mmHg (mediana, IIQ)	120 (100-160)	120 (90-166)	0,9
DBP, mmHg (mediana, IIQ)	77,5 (60-98)	72 (60-100)	0,7
HR, bpm (média ± DP)	64 (51-76)	63 (40-67)	0,09
CA, cm (média ± DP)	98,9 ± 9,1	104,8 ± 11,7	0,08
IMC, Kg/m^2^ (média ± SD)	29,7 ± 4,6	29,9 ± 4,1	0,9
DAC de padrão obstrutivo (%)			0,1
Doença de único vaso	0	13	
Doença de dois vasos	9,1	8,7	
Doença de três vasos	90,9	78,3	
FEVE no ecocardiograma, % (média ± DP)	54,4 ± 7,6	49,9 ± 9,9	0,09
**História médica**			
Tempo desde o diagnóstico de DAC, anos (média ± DP)	11,9 ± 7,2	12,0 ± 9,6	0,9
Angioplastia prévia (%)	77,2	43,4	0,02
Cirurgia de revascularização do miocárdio (CABG)%	90,0	69,5	0,07
Acidente vascular cerebral (AVC) prévio (%)	4,5	2,2	0,5
Infarto do miocárdio prévio (%)	68,2	77,8	0,2
Diabetes mellitus (%)	59,1	82,6	0,08
Hipertensão (%)	72,7	87	0,3
Tabagismo (atual ou anterior) (%)	54,5	68,8	0,1
Hiperlipidemia (%)	95,5	93,3	0,9
Obesidade (%)	31,8	35,6	0,6
Inatividade física (%)	72,7	75,6	0,7
Histórico familiar de DAC (%)	61,9	60,9	0,9
**Achados laboratoriais**			
Hemoglobina, g/dL (média ± DP)	14,0 ± 1,5	13,3 ± 1,5	0,1
Creatinina, mg/dL (média ± DP)	0,9 ± 0,2	1,2 ± 0,6	0,06
Colesterol LDL, mg/dL (média ± DP)	75,0 ± 25,4	89,9 ± 40,8	0,2
Colesterol HDL, mg/dL (média ± DP)	39,7 ± 9,0	47,3 ± 12,6	0,03
Triglicerídeos, mg/dL (média ± DP)	143,1 ± 42,6	112,8 ± 41,0	0,02
Glicemia de jejum, mg/dL (mediana, IIQ)	125,5 (99-207)	118 (91-370)	0,9
Hemoglobina glicada, % (mediana, IIQ)	6,3 (5,4-10,1)	6,2 (5,5-12,4)	0,9
PCR, mg/dL (mediana, IQR)	0,9 (0,1-11,1)	1,4 (0,4-9,2)	0,1

GR: grupo de reabilitação; GC: grupo controle; CCS: Canadian Cardiovascular Society; AAW: ataques de angina por semana; SANCW: consumo de nitrato de ação rápida por semana; PAS: Pressão Arterial Sistólica; PAD: Pressão Arterial Diastólica; FC: frequência cardíaca; DP: desvio padrão; CA: circunferência abdominal; IMC: índice de massa corporal; DAC: doença arterial coronariana; FEVE: fração de ejeção do ventrículo esquerdo; CABG: cirurgia de revascularização do miocárdio (bypass coronário); LDL: lipoproteína de baixa densidade; HDL: lipoproteína de alta densidade; IIQ: intervalo interquartil; PCR: proteína c reativa. IIQ: intervalo interquartil.

A complexidade dos pacientes foi evidente em ambos os grupos, como demonstrado pelas altas taxas de fatores de risco cardiovascular, padrões anatômicos da DAC, condições coexistentes e pelo uso frequente de múltiplos agentes antianginosos ( Tabela 3 ). Os achados laboratoriais não revelaram diferenças significativas entre os grupos, exceto pelos níveis de HDL-colesterol e triglicerídeos ( Tabela 2 ).

**Tabela 3:** – Medicamentos usados pelos pacientes no basal

Medicamento	GR (n = 22)	GC (n = 23)	Valor p
**Aspirina**			
Pacientes (%)	95,5	95,7	>0,9
**Clopidogrel**			
Pacientes (%)	40,9	17,4	0,08
**Estatinas**			
Pacientes (%)	100	100	1,0
**Ezetimibe**			
Pacientes (%)	9,1	17,4	0,7
**Betabloqueadores**			
Pacientes (%)	95,5	100	0,5
% dosagem máxima (mediana, IIQ)	100 (25-100)	75 (12,5-100)	0,6
**Bloqueadores de canais de cálcio**			
Pacientes (%)	90,9	87	>0,9
% dosagem máxima (mediana, IIQ)	100 (25-100)	100 (50-100)	
**Nitratos de longa duração**			
Pacientes (%)	90,9	95,7	0,6
% dosagem máxima (mediana, IIQ)	100 (50-100)	100 (50-100)	0,3
**Trimetazidina**			
Pacientes (%)	95,5	95,7	>0,9
**Ivabradina**			
Pacientes (%)	18,2	13	0,7
**Inibidores de ECA**			
Pacientes (%)	40,9	60,9	0,2
% dosagem máxima (mediana, IIQ)	50 (25-100)	100 (33-100)	0,3
**BRAs**			
Pacientes (%)	22,7	30,4	0,6
% dosagem máxima (mediana, IIQ)	100 (50-100)	100 (50-100)	0,4
**Diuréticos**			
Pacientes (%)	36,4	47,8	0,4
**Agentes antidiabéticos orais**			
Pacientes (%)	50,0	52,2	0,9
**Insulina**			
Pacientes (%)	27,3	30,4	0,8

GR: grupo de reabilitação; GC: grupo controle; ECA: enzima conversora de angiotensina; BRAs: bloqueadores dos receptores de angiotensina; IIQ: Intervalo Interquartil.

### Efeitos do treinamento físico

Dezenove pacientes do grupo RG completaram o protocolo; dois pacientes desistiram por motivos pessoais e um devido a um evento cardiovascular. A adesão ao programa de treinamento físico foi de 76,7 ± 19,0% das sessões.

A duração média do TA foi de 39,3 ± 3,7 minutos. A FC média durante o TA foi de 78,7 ± 5,4 bpm, correspondendo a 50,1 ± 4,3% da FC máxima estimada pela idade e 96,7 ± 11,3% da FC no limiar ventilatório 1 (VT1). A FC limiar para angina durante o TA foi de 81,3 ± 8,9 bpm, próxima ao LA/isquemia identificado no TCPE. Quinze pacientes (73,7%) apresentaram angina durante o TA, e cinco deles (26,3%) precisaram utilizar nitrato sublingual em pelo menos uma sessão. A pressão arterial sistólica média e a pressão arterial diastólica média durante o TA foram de 121,7 ± 14,1 mmHg e 70,8 ± 5,6 mmHg, respectivamente.

Apesar da presença de angina, não ocorreram arritmias ou depressões do segmento ST que exigissem a interrupção do exercício durante o TA.^20^ Em relação ao treinamento resistido, os pacientes não relataram sintomas de angina e/ou qualquer desconforto muscular; portanto, o treinamento resistido foi realizado conforme o planejado.

### Avaliação clínica

Após 12 semanas de protocolo, não foram observadas diferenças significativas entre os grupos quanto à classe funcional da angina segundo a CCS, AAW, SANCW ou parâmetros clínicos e laboratoriais ( Tabela 4 ).

**Tabela 4:** – Alterações nos parâmetros clínicos em cada grupo

Variável	GR		GC		Valor p		
	Pré	Pós	Pré	Pós	G	M	Int.
**Dados clínicos**							
Classe funcional da angina CCS, n					0,81	0,01	0,26
0		1 (5,3)					
I		6 (31,6)		3 (14,3)			
II	7 (31,8)	6 (31,6)	10 (43,5)	9 (42,9)			
III	6 (27,3)	1 (5,3)	5 (21,7)	3 (14,3)			
IV	9 (40,9)	5 (26,3)	8 (34,8)	6 (28,6)			
Frequência de angina, n	11,6±10,1	7,18±12,5	8,8±11,1	10,4±11,1	0,76	0,26	0,05
Consumo de nitrato, n	4,6±6,5	4,5±6,9	4,7±7,7	5,8±9,1	0,76	0,51	0,55
PAS, mmHg	124±16,8	119±16,5	123±17,6	121±20,2	0,87	0,26	0,70
PAD, mmHg	76,3±9,4	72,3±9,6	74,9±12,0	72,5±12,7	0,83	0,10	0,68
FC, bpm	63,3±6,5	61,7±5,7	59,3±7,4	63,3±9,6	0,51	0,25	0,02
IMC, kg/m^2^	29,7±4,6	29,8±5,4	29,9±4,1	28,7±4,2	0,71	0,78	0,31
**Dados laboratoriais**							
Glicemia de jejum, mg/dL	131±31,5	123±26,8	149±72,8	143±82,8	0,22	0,53	0,66
HBA1C, %	6,7±1,3	6,5±0,9	7,0±1,8	6,8±1,6	0,35	0,76	0,37
LDL-c, mg/dL	75,1±25,4	72,8±23,2	89,9±40,9	93,2±42,9	0,145	0,56	0,94
HDL-c, mg/dL	39,7±9,0	40,0±9,9	47,3±12,7	48,1±10,3	0,033	0,79	0,25
TG, mL/g	143±42,6	136±50	113±41	141±71,1	0,331	0,18	0,01
P, mg/dL	1,68±2,3	1,8±2,7	2,7±2,6	2,1±2,0	0,355	0,86	0,42

Os dados são expressos como média ± desvio padrão ou número (%). GR: Grupo de Reabilitação; GC: grupo controle G: grupo M: momento Int.: Interação Pré: antes da reabilitação ou do tratamento médico isolado; Pós: após a reabilitação ou o tratamento médico isolado; CCS: Canadian Cardiovascular Society; PAS: Pressão Arterial Sistólica; PAD: Pressão Arterial Diastólica; FC: frequência cardíaca; IMC: Índice de Massa Corporal; HBA1C: hemoglobina glicada; LDL: lipoproteína de baixa densidade; HDL: lipoproteína de alta densidade CRP: proteína C reativa; G: RG vs CG M: pré vs pós Int.: efeito combinado entre grupo e momento.

### Avaliação por EEE

**Figura 2:**
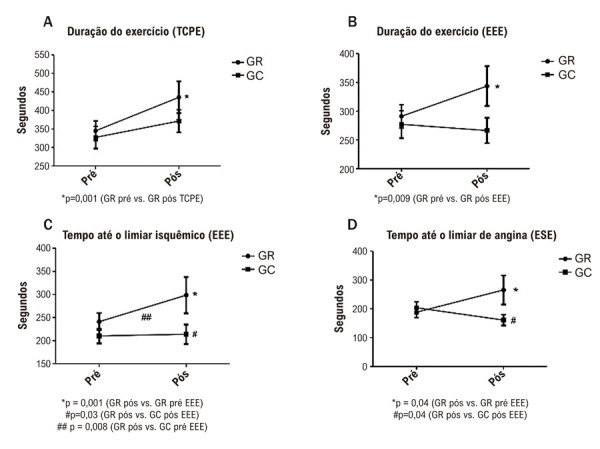
– Alteração na duração do exercício no teste cardiopulmonar de exercício, duração do exercício, tempo até o limiar isquêmico e tempo até o limiar de angina na ecocardiografia de estresse com exercício, por grupo de estudo; GR: grupo de reabilitação; GC: grupo controle; EEE: ecocardiografia de estresse com exercício.

Houve uma melhora significativa na carga máxima, na quantificação da angina, na duração do exercício e no tempo até os limiares de angina e isquemia entre os grupos, conforme mostrado na Tabela 5 e nas Figuras 2B, 2C e 2D , respectivamente. Não foram encontradas diferenças na FEVE média, nos escores isquêmicos, na FC máxima ou na FC nos LI e LA ( Tabelas 5 e 6 ).

**Tabela 5:** – Alterações nos parâmetros ecocardiográficos e cardiopulmonares em cada grupo

Variável	GR		GC		Valor p		
	Pré	Pós	Pré	Pós	G	M	Int,
**EEE**							
FEVE, %	54,5±7,6	53±7,7	50±9,9	48,8±9,9	0,06	0,03	0,67
Escore em repouso, n	1,28±0,22	1,28±0,16	1,45±0,37	1,37±0,28	0,06	0,51	0,35
Escore no exercício, n	1,49±0,28	1,36±0,21	1,62±0,38	1,49±0,29	0,08	<0,01	0,62
Delta score, ua	0,21±0,16	0,08±0,11	0,16±0,11	0,12±0,11	0,95	<0,01	0,10
Duração do exercício, s	291±89,5	344±143	277±110	266±93,7	0,07	0,02	<0,01
Carga máxima (W), J/s	34,7±17,8	46,2±23,8	35,2±24,2	35±21,4	0,31	<0,01	<0,01
Quantificação da angina, n	5,2±3,2	4,4±2,6	6,4±2,3	6,6±2,5	0,01	0,04	<0,01
Pico (FC), bpm	96,1±9,7	95,5±18	99,5±15,8	94,8±11,9	0,84	0,58	0,07
LI (FC), bpm	90,5±7,2	93,5±12,7	93,6±15,7	91,6±10,5	0,93	0,88	0,20
Tempo para LI, s	241±86,4	299±136	210±74,9	214±83,4	0,02	<0,01	<0,01
LA (FC), bpm	89,1±7,5	89,2±8,9	92,1±16,5	90,6±10,2	0,60	0,70	0,65
LA (tempo), s	188±80,6	265±160	204±95,2	161±69,8	0,09	0,12	<0,01
**TCPE**							
Pico VO_2_ mL.kg.min^-1^	15,9±3,3	16,3±4,3	15,7±3,6	15,6±3,5	0,51	0,72	0,46
VO_2_ previsto, %	63,8±19,2	64,5±18,3	60,2±14,8	61,3±10,3	0,31	0,80	0,78
Quantificação da angina, n	6,4±1,5	6,5±1,8	6,2±2,0	6,4±2,7	0,95	0,85	0,66
VT1 (HR), bpm	82,1±9,6	82,7±9,7	87,6±16,0	83,6±10,5	0,34	0,45	0,37
LA (time), s	198±66	322±174	248±132	287±94	0,86	<0,05	0,07
LA (HR), bpm	85,9±13,9	88±13,1	92,5±16,3	90,7±15,2	0,33	0,95	0,36
QR, ua	0,9±0,99	1,0±0,1	1,0±0,1	1,0±0,1	0,93	0,74	0,01
Oxigênio de pulso, mL/batimento	13,6±3,7	14,7±3,4	12:3±3:1	12,9±3,6	0,18	<0,01	0,43
Distância percorrida, m	312±151	443±205	360±155	363±130	0,76	<0,01	<0,01
Duração total, s	345±124	435±181	327±141	371±128	0,18	<0,01	<0,01

Os dados são apresentados como média ± desvio padrão. GR: Grupo de Reabilitação; GC: grupo controle; G: Grupo; M: Momento; Int.: Interação; Pré: antes da reabilitação ou do tratamento médico isolado; Pós: após a reabilitação ou o tratamento médico isolado; EEE: ecocardiografia de estresse com exercício; FEVE: fração de ejeção do ventrículo esquerdo; FC: frequência cardíaca; LI: limiar isquêmico; LA: limiar de angina; TCPE: teste cardiopulmonar de exercício VT1: primeiro limiar ventilatório; QR: Quociente Respiratório; G: GR vs GC; M: pré vs. pós; Int.: efeito combinado entre grupo e momento.

**Tabela 6:** – Tamanho do efeito (d de Cohen) e Intervalo de Confiança (IC)

Variáveis (pós-GR vs. pós-GC)	d de Cohen	IC 95% Inferior		IC 95% Superior	G	M	Int,
EEE							
Duração do exercício, s	0,65	-5,79	186,32		0,07	0,02	<0,01
Carga máxima (W), J/s	0,49	-7,18	31,76		0,31	<0,01	<0,01
Quantificação da angina, n	0,87	-5,58	-0,63		0,01	0,04	<0,01
Tempo para LI, s	0,80	5,79	191,09		0,02	<0,01	<0,01
Tempo par LA, s	0,96	0,27	223,25		0,09	0,12	<0,01
TCPE							
Distância percorrida, m	0,47	-66,45	225,76		0,76	<0,01	<0,01
Duração total, s	0,41	-32,31	231,41		0,18	<0,01	<0,01

Pós-GR: grupo de reabilitação após o período de reabilitação; pós-GC: grupo controle após tratamento médico isolado; G: grupo M: momento Int.: interação EEE: Ecocardiografia de Estresse com Exercício; LI: limiar isquêmico; LA: limiar de angina TCPE: Teste Cardiopulmonar de Exercício; G: GR vs. GC M: pré vs pós Int.: efeito combinado entre grupo e momento.

### Avaliação de desempenho no TCPE

Houve um aumento significativo na duração do exercício e na distância percorrida durante o TCPE no grupo RG, conforme demonstrado na Tabela 5 e na Figura 2A . Nenhuma diferença significativa foi observada no consumo máximo de oxigênio (VO_2_ pico) ou em outras variáveis do TCPE ( Tabelas 5 e 6 ).

## Discussão

Pela primeira vez, a segurança e a eficácia da RCE na melhora dos sintomas, da carga isquêmica e da capacidade funcional foram avaliadas objetivamente em pacientes com AR. Nossos resultados demonstram que um programa de ECR de 12 semanas, realizado próximo aos LA e/ou VT1, foi seguro e apresentou efeitos positivos na duração do exercício, intensidade da angina e nos limiares isquêmico e de angina durante a EEE.

Desde que a RA foi documentada pela primeira vez como uma condição clínica no início dos anos 2000, a ECR tem sido considerada uma opção de tratamento. No entanto, ainda faltam evidências robustas que sustentem sua segurança e orientem sua aplicação no contexto da AR.^6 , 14 , 15 , 26 , 27^

Em nosso estudo, os pacientes apresentavam angina debilitante devido à isquemia miocárdica, apesar do uso de pelo menos três classes de agentes antianginosos, e não eram elegíveis para intervenção coronariana devido à complexidade da doença coronariana obstrutiva. Consequentemente, esses pacientes apresentavam um perfil de alto risco para eventos cardíacos adversos induzidos pelo exercício,^8^ e suas limitações físicas tornaram tanto a prescrição quanto a execução segura do exercício particularmente desafiadoras – especialmente para alcançar a intensidade moderada-alvo.

No entanto, o treinamento físico guiado por parâmetros do TCPE e pelo LA – permitindo a manifestação de sintomas de angina até o nível 3 em uma escala de 0 a 10 – não aumentou o risco de eventos cardiovasculares durante o período do estudo. Esses dados são reforçados por resultados anteriores, que demonstraram que uma sessão aguda de exercício aeróbico não altera os níveis de hs-cTnT em pacientes com AR, sugerindo que nenhum dano miocárdico significativo foi provocado quando o exercício foi realizado conforme o protocolo prescrito.^15^

Apesar da ausência de melhora nos parâmetros clínicos definidos pela classe funcional da angina (CCS), AAW e SANCW – variáveis autorreferidas – consideramos a melhora nos parâmetros dos testes de exercício um indicador fundamental do efeito positivo da RCE. O aumento na duração do exercício, a redução na intensidade da angina e o atraso no início dos sintomas podem impactar positivamente a qualidade de vida, que é o foco central do manejo da AR. Além disso, os parâmetros dos testes de exercício são medidas objetivas, tornando-os indicadores confiáveis de melhora real em pacientes com RA na prática clínica.

Não foi observada melhora significativa no VO_2_ pico. Devido às limitações físicas decorrentes do baixo LA, manter a intensidade moderada-alvo no TA foi desafiador – e, por vezes, inviável – o que pode explicar a ausência de melhora no VO_2_ pico. Ainda assim, mesmo sem aumento no VO_2_ pico (o parâmetro prognóstico mais importante no TCPE), o aumento na distância percorrida durante o teste de exercício pode ser interpretado como uma melhora clínica, já que também é considerado um marcador prognóstico em pacientes com doença cardíaca.^28^

A melhora no desempenho durante o exercício (sem alteração concomitante no VO_2_ pico) pode ser parcialmente explicada por adaptações iniciais, como alterações neurológicas e musculares induzidas pelo treinamento resistido.^10 , 29 , 30^

Portanto, a RC é uma terapia adjuvante viável para pacientes com AR, corroborando sua indicação clínica. Acreditamos que o programa de RCE de 12 semanas representa um ponto de partida para os benefícios do exercício em pacientes com RA, e que a continuidade da RCE pode promover efeitos adicionais positivos e melhorar a qualidade de vida.

### Limitações

Os resultados desta análise devem ser interpretados considerando as possíveis limitações:

O cálculo do tamanho da amostra foi baseado no único estudo anterior que avaliou o efeito da RC em pacientes com AR, apesar de apresentar desfechos pré-determinados diferentes.

O estudo apresentou dificuldades de inclusão devido aos critérios de inclusão rigorosos.

## Conclusão

Um programa de RCE de 12 semanas foi seguro e eficaz na melhora da capacidade funcional e da carga isquêmica avaliada pelo EEE em pacientes com AR.
