# Supplementary material for: Segurança e Eficácia da Reabilitação Cardíaca Baseada em Exercício em Pacientes com Angina Refratária
Source: Arq Bras Cardiol. 2026 Jan 9;122(12):e20250331. [Article in Portuguese] doi: 10.36660/abc.20250331 (PMC12978375; doi:10.36660/abc.20250331)
Supplement: Translation

# Translation: Safety and Efficacy of Exercise-based Cardiac Rehabilitation in Patients with Refractory Angina

**DOI:** 10.36660/abc.20250331i

**Authors:** Luciana Oliveira Cascaes Dourado, Camila Paixão Jordão, Marcelo Luiz Campos Vieira, Luis Henrique Wolff Gowdak, Carlos Eduardo Negrão, Luiz Antonio Machado Cesar, Luciana Diniz Nagem Janot de Matos

**Affiliations:** 1 Brazil Instituto do Coração do Hospital das Clínicas da Faculdade de Medicina da Universidade de São Paulo, São Paulo, SP – Brazil; 2 Brazil Hospital Israelita Albert Einstein, São Paulo, SP – Brazil

**Keywords:** Angina, Cardiac Rehabilitation, Myocardial Ischemia

## Abstract

**Background:**

Evidence on the safety and anti-ischemic effects of exercise-based cardiac rehabilitation (ECR) in patients with refractory angina (RA) remains limited.

**Objective:**

To evaluate the safety and efficacy of a 12-week ECR program in patients with RA, focusing on improvements in symptoms, functional capacity, and ischemic burden assessed by exercise stress echocardiography (ESE).

**Methods:**

This was a prospective, single-center, randomized controlled trial evaluating a 12-week ECR program in patients with RA. Forty-five patients were randomized to either the rehabilitation group (RG), receiving ECR, or the control group (CG), receiving medical treatment (MT) alone. Outcomes included mortality, cardiovascular events, anginal symptoms, and parameters from ESE and cardiopulmonary exercise testing (CPET). Statistical significance was set at p < 0.05.

**Results:**

In ESE, exercise duration was significantly greater in RGpost (after ECR) compared to RGpre (before ECR) (∆ = 63.24 ± 19.87 s; p < 0.01). Angina quantification was lower in RGpost than in RGpre, CGpost (after MT alone), and CGpre (before MT alone) (∆ = –1.64 ± 0.48 n, p < 0.01; –3.10 ± 0.97 n, p < 0.01; and –2.73 ± 0.92 n, p = 0.01, respectively). The angina threshold was higher in RGpost than in RGpre and CGpost (∆ = 89.66 ± 33.16 s, p = 0.04; and 111.76 ± 42.25 s, p = 0.04, respectively). Improvement in ischemic burden on ESE was demonstrated by increased time to ischemic threshold in RGpost compared to RGpre, CGpost, and CGpre (∆ = 83.23 ± 21.84 s, p < 0.01; 98.44 ± 35.11 s, p = 0.03; and 109.34 ± 34.00 s, p < 0.01, respectively). In CPET, RGpost showed increased exercise duration (∆ = 104.54 ± 28.09 s, p < 0.01) and distance covered (∆ = 131.23 ± 30.48 m, p < 0.01) compared to RGpre. No significant differences in VO_2_were observed between groups. Two patients in the CG group died. One patient in the RG group experienced prolonged angina during training. No significant differences in major cardiovascular events were observed between groups.

**Conclusion:**

The 12-week ECR-program was safe and effective in improving exercise duration, distance covered and ischemic burden on ESE in patients with RA.

## Introduction

Refractory angina (RA) is a chronic and debilitating condition lasting more than three months, characterized by limiting angina due to myocardial ischemia that is not controlled by a combination of medical therapy, angioplasty, and surgery.^1 , 2^Patients with RA experience significant impairment in quality of life,^3^and several promising alternative therapies – some experimental – have been tested with varying results.^4 , 5^

Therefore, there is an urgent need to incorporate safe, effective, and accessible therapies to alleviate symptoms in RA. Exercise-based cardiac rehabilitation (ECR) is considered the standard of care for the prevention and treatment of chronic coronary artery disease (CAD).^6 - 8^It promotes beneficial cardiovascular adaptations that improve cardiorespiratory fitness,^9^exert anti-ischemic effects,^10 , 11^and reduce cardiovascular events and mortality.^12^

Despite its benefits, ECR is not frequently prescribed to patients with RA in clinical practice due to concerns about adverse events during physical exercise.^1 3^This hesitation stems from the limited evidence supporting its safety and efficacy in the context of RA,^14 , 15^as these patients typically have a low ischemic threshold and significant limitations in physical performance.^6 , 8 , 13 , 16^

Previous studies have shown that moderate exercise training in patients with RA can improve functional capacity – measured by Progressive Shuttle Walk test performance – and reduce angina threat perception, although it does not appear to enhance perceived quality of life.^14^Nevertheless, little is known about the safety and anti-ischemic effects of ECR in this population, particularly regarding improvements in anginal symptoms, functional capacity, and ischemic burden as assessed by exercise stress echocardiography (ESE), when patients are treated with contemporary medical therapy and clinically appropriate revascularization strategies.

In this study, we tested the hypothesis that a 12-week ECR program is a safe and effective strategy to improve anginal symptoms, functional capacity, and ischemic burden as assessed by ESE in patients with RA.

## Methods

### Study design

This was a prospective, single-center, randomized controlled trial evaluating a 12-week cardiac rehabilitation program in patients with RA. The study was conducted at a public tertiary university hospital in São Paulo, Brazil (see Central Illustration ). It was approved by the local ethics and research committee (CAAE: 24308213.7.0000.0068) and registered at ClinicalTrials.gov (NCT03218891). The study adhered to the principles outlined in the Declaration of Helsinki. All participants provided written informed consent.

### Study patients

Patients diagnosed with RA during clinical follow-up at a specialized outpatient clinic were enrolled. Eligible participants were of both sexes, aged 45 to 75 years, with angina (Canadian Cardiovascular Society [CCS] functional class II to IV) persisting for more than three months despite optimal medical therapy (≥3 antianginal medications). Included patients had documented myocardial ischemia confirmed by exercise stress echocardiography (ESE) and were not eligible for surgical or percutaneous myocardial revascularization procedures.

### Exclusion criteria

Exclusion criteria included: 1) presence of permanent pacemakers or implantable cardioverter-defibrillators; 2) non-sinus cardiac rhythm; 3) history of acute coronary syndrome or myocardial revascularization (surgical or percutaneous) within the past three months; 4) functional impairment due to any clinical condition that precludes exercise; 5) activity restriction classified as Class D according to the American Heart Association criteria for risk stratification of exercise-related events.^17 , 18^

### Randomization and intervention

Patients who met the inclusion criteria were randomized in blocks to ensure ample homogeneity given the sample size. A computer-generated sequence was used to assign them to one of two groups: 1) ECR group (RG), consisting of optimized clinical treatment combined with a 12-week physical training program or 2) control group (CG), consisting of optimized medical treatment alone. All patients underwent clinical and laboratory evaluations, cardiopulmonary exercise testing (CPET), and ESE both at baseline and at the end of the study.

### Study endpoints

The prespecified endpoints included evaluating the safety of a 12-week ECR program in patients with RA. This assessment focused on clinical events (death and major cardiovascular events), as well as the impact on anginal symptoms – angina functional class, angina attacks *per* week (AAW) and short-acting nitrate consumption *per* week (SANCW). Additional endpoints included myocardial ischemic burden evaluated through ESE, and functional capacity evaluated through CPET and ESE.

### Clinical evaluation

Patients were assessed during medical visits both before and after the intervention by a cardiologist who was blinded to group allocation. The CCS angina functional class was evaluated. The AAW and SANC were recorded in an angina diary. Adherence to medical therapy was encouraged during visits, based on each patient’s tolerance. Antianginal medications were prescribed in accordance with current clinical guidelines.^7^

### Cardiopulmonary exercise testing (CPET)

To verify effectiveness and prescribe the aerobic exercise training (AET), CPET was performed both before and after the intervention using a motorized treadmill (T2100 Model, GE Healthcare, USA) and an ergospirometer (SensorMedics – VmaxAnalyzer Assembly, Encore 29S, USA), following a graded exercise protocol (modified Balke 2.5 mph). CPET was performed in accordance with the American Heart Association guidelines.^19^The exercise test was performed, interpreted and interrupted according to the Brazilian Guidelines of the exercise test.^20^CPET was evaluated using cardiopulmonary fitness parameters, and also through angina exercise threshold and its quantification. Angina threshold (AT) was determined at the exact time (in seconds) and HR at which the patient complained of angina symptoms.

### Exercise stress echocardiography (ESE)

To assess myocardial ischemic burden, a two-dimensional echocardiographic evaluation was performed with the Vivid9 device (version 110.x.x, GE Healthcare), in accordance with the guidelines of the American Society of Echocardiography.^21^Myocardial ischemic burden on ESE was defined by 1) ischemic score, calculated as the difference between stress and rest scores, and/or 2) ischemic threshold (IT), determined during ESE. Exercise testing was performed on a lower limb cycle ergometer, with workload progressively increased from 5 to 25 J/s every 3 minutes. The test was interrupted based on guidelines criteria.^21 , 22^Segmental contractility was assessed using a 17-segment model, and the wall motion score index was defined based on established guidelines.^21^The onset of ischemia identified at the time and HR when regional myocardial wall motion abnormalities first appeared, which was considered the IT. ESE functional capacity was evaluated through exercise duration, peak load, AT (HR and time) and angina quantification. Angina was graded using a subjective pain scale ranging from 0 (no pain) to 10 (very intense).^23^

### Exercise-based cardiac rehabilitation

The ECR program was conducted at a cardiovascular rehabilitation center in a tertiary hospital, within a temperature-controlled training room equipped with stationary bikes, treadmills, and strength training equipment, and supervised by qualified rehabilitation professionals. The protocol consisted of 36 exercise sessions over 12 weeks, with sessions held three times per week. Each supervised session lasted 60 minutes and included: 40 minutes of AET, 15 minutes of resistance training, and 5 minutes of stretching. The AET component comprised a 5-minute warm-up, 30 minutes of continuous aerobic exercise on a motorized treadmill, and a 5-minute cool-down. AET prescription was guided by exercise-related physiological parameters identified during CPET,^24^targeting a heart rate (HR) corresponding to at least the first ventilatory threshold (VT1) and/or the AT, in cases where AT HR was below VT1 HR.

Continuous exercise was encouraged; however, if the patient experienced mild to moderate angina (rated up to 3 on a pain scale from 0 [no pain] to 10 [severe pain]), brief interruptions or a reduction in intensity were recommended.^23^Exercise was resumed once symptoms subsided. Patients were continuously monitored via telemetry, and HR was recorded at the midpoint and end of each aerobic session to calculate the mean training HR. Sublingual isosorbide dinitrate (5 mg) was administered as needed, based on individual angina tolerance. In such cases, patients were advised not to resume exercise. Resistance exercise sessions followed the latest guidelines.^6 , 25^Moderate intensity was targeted, based on the perceived exertion scale (levels 4 to 6), and consisted of two sets of 8 to 15 repetitions. Exercises were performed rhythmically, avoiding breath-holding and straining (Valsalva maneuver), with exhalation during the contraction or exertion phase and inhalation during the relaxation phase.^25^

### Statistical analysis and sample size calculation

Data were analyzed using SPSS Statistics 20 software. Continuous variables were expressed as mean ± standard deviation (SD) and categorical variables as percentages. Continuous variables that did not present a normal distribution were described using median and interquartile range. The Kolmogorov Smirnov test was used to assess data distribution. For comparison between groups, was applied Student’s t-test unpaired or Mann-Whitney according to distribution for continuous variables, or the Chi-square test for categorical variables, as appropriate. Generalized Estimating Equations (GEE) were used for longitudinal data analysis, and Bonferroni post hoc tests were conducted when significant differences were identified. Statistical significance was set at a p < 0.05.

To determine the sample size, we based on a previous study^14^assessing functional capacity using Shuttle Walking Test, assuming a standard deviation of 1.2 and a mean difference among groups was 0.96 between groups. Using these parameters, we calculated that 26 participants were required in each group (intervention and control) to achieve 80% statistical power at a 5% significance level. The sample size calculation was performed using the Power and Sample Size Calculator (PS), version 3.0.43.

The effect size for the continuous outcomes was calculated as the difference in estimated marginal means divided by the model-based standard deviation, a metric analogous to Cohen’s d. This approach accounts for the within-subject correlation in the longitudinal data.

## Results

The flow diagram ( Figure 1 ) illustrates group allocation, withdrawals, and follow-up. A total of 45 patients were included in the study – 22 assigned to the RG and 23 to the CG.

**Figure 1:** – Flow diagram of rehabilitation group versus control group.

### Safety evaluation

Two patients in the CG died during the study – 1 due to cardiovascular causes. One patient in the RG experienced prolonged angina during a training session, was admitted to the emergency department for observation, and subsequently withdrawn from the study. No significant differences in major cardiovascular events were observed between the groups at the end of the study ( Table 1 ).

**Table 1:** – Cardiovascular events during the study period

Events	RG (n=22)	CG (n=23)	p value
Death, n (%)	0	2 (8.7)	0.5
Cardiovascular death, n (%)	0	1 (4.3)	> 0.9
Cardiovascular major events, n (%)	1 (4.5)	0	0.5

RG: rehabilitation group; CG: control group.

### Baseline characteristics

There were no significant clinical differences between the groups at baseline ( Table 2 ) with respect to CCS angina functional class, AAW, SANCW, and other clinical parameters. Both groups had a normal mean left ventricular ejection fraction (LVEF). Patients in the RG group had undergone more coronary interventions than those in the CG group.

**Table 2:** – Demographic and clinical characteristics of patients at baseline

Baseline patient data	RG (n=22)	CG (n=23)	p value
Age, years (mean ± SD)	61.4±7.9	60.7±9.2	0.8
Male (%)	68.2	60.9	0.6
**Clinical findings**			
CCS angina functional class (%)			0.7
II	31.8	43.5	
III	27.3	21.7	
IV	40.9	34.8	
AAW, n (median, IQR)	7 (1 - 35)	4 (0.5 - 49)	0.1
SANCW, n (median, IQR)	1.25 (0 – 24.5)	1 (0 – 35)	0.4
SBP, mmHg (median, IQR)	120 (100-160)	120 (90-166)	0.9
DBP, mmHg (median, IQR)	77.5 (60-98)	72 (60-100)	0.7
HR, bpm (mean ± SD)	64 (51-76)	63 (40-67)	0.09
AC, cm (mean ± SD)	98.9 ± 9.1	104.8 ± 11.7	0.08
BMI, kg/m^2^(mean ± SD)	29.7 ± 4.6	29.9 ± 4.1	0.9
CAD obstructive pattern (%)			0.1
One-vessel disease	0	13	
Two-vessel disease	9.1	8.7	
Three-vessel disease	90.9	78.3	
Echocardiographic LVEF, % (mean ± SD)	54.4 ± 7.6	49.9 ± 9.9	0.09
**Past medical history**			
Time from diagnosis of CAD, years (mean ± SD)	11.9 ± 7.2	12.0 ± 9.6	0.9
Previous angioplasty (%)	77.2	43.4	0.02
Previous CABG (%)	90.0	69.5	0.07
Previous stroke (%)	4.5	2.2	0.5
Previous myocardial infarction (%)	68.2	77.8	0.2
Diabetes mellitus (%)	59.1	82.6	0.08
Hypertension (%)	72.7	87	0.3
Smoking (previous or current) (%)	54.5	68.8	0.1
Hyperlipidemia (%)	95.5	93.3	0.9
Obesity (%)	31.8	35.6	0.6
Physical inactivity (%)	72.7	75.6	0.7
Family history of CAD (%)	61.9	60.9	0.9
**Laboratory findings**			
Hemoglobin, g/dL (mean ± SD)	14.0 ± 1.5	13.3 ± 1.5	0.1
Creatinine, mg/dL (mean ± SD)	0.9 ± 0.2	1.2 ± 0.6	0.06
LDL-cholesterol , mg/dL (mean ± SD)	75.0 ± 25.4	89.9 ± 40.8	0.2
HDL-cholesterol, mg/dL (mean ± SD)	39.7 ± 9.0	47.3 ± 12.6	0.03
Triglycerides, mg/dL (mean ± SD)	143.1 ± 42.6	112.8 ± 41.0	0.02
Fasting glucose, mg/dL (median, IQR)	125.5 (99-207)	118 (91-370)	0.9
Glycated hemoglobin, (%) (median, IQR)	6.3 (5.4-10.1)	6.2 (5.5-12.4)	0.9
CRP, mg/dL (median, IQR)	0.9 (0.1-11.1)	1.4 (0.4-9.2)	0.1

RG: rehabilitation group; CG: control group; CCS: Canadian Cardiovascular Society; AAW: angina attacks per week; SANCW: short-acting nitrate consumption per week; SBP: systolic blood pressure; DBP: diastolic blood pressure; HR: heart rate; SD: standard deviation; AC: abdominal circumference; BMI: body mass index; CAD: coronary artery disease; LVEF: left ventricular ejection fraction; CABG: Coronary Artery Bypass Graft; LDL: low density lipoprotein; HDL: high density lipoprotein; IQR: interquartile range; CRP: C-reactive protein.

Patient complexity was evident in both groups, as demonstrated by high rates of cardiovascular risk factors, coronary artery disease (CAD) anatomical patterns, coexisting conditions, and the frequent use of multiple antianginal agents ( Table 3 ). Laboratory findings revealed no significant differences between the groups, except for HDL-cholesterol and triglyceride levels ( Table 2 ).

**Table 3:** – Baseline medications used by patients allocated in each group

Medication	RG (n = 22)	CG (n = 23)	p value
**Aspirin**			
Patients (%)	95.5	95.7	>0.9
**Clopidogrel**			
Patients (%)	40.9	17.4	0.08
**Statins**			
Patients (%)	100	100	1.0
**Ezetimibe**			
Patients (%)	9.1	17.4	0.7
**β-Blockers**			
Patients (%)	95.5	100	0.5
% maximum dosage (median, IQR)	100 (25-100)	75 (12.5-100)	0.6
**Calcium channel blockers**			
Patients (%)	90.9	87	>0.9
% maximum dosage (median, IQR)	100 (25-100)	100 (50-100)	
**Long-acting nitrates**			
Patients (%)	90.9	95.7	0.6
% maximum dosage (median, IQR)	100 (50-100)	100 (50-100)	0.3
**Trimetazidine**			
Patients (%)	95.5	95.7	>0.9
**Ivabradine**			
Patients %	18.2	13	0.7
**ACE inhibitors**			
Patients %	40.9	60.9	0.2
% maximum dosage (median, IQR)	50 (25-100)	100 (33-100)	0.3
**ARBs**			
Patients (%)	22.7	30.4	0.6
% maximum dosage (median, IQR)	100 (50-100)	100 (50-100)	0.4
**Diuretics**			
Patients (%)	36.4	47.8	0.4
**Oral antidiabetic agents**			
Patients (%)	50.0	52.2	0.9
**Insulin**			
Patients (%)	27.3	30.4	0.8

RG: rehabilitation group; CG: control group; ACE: angiotensin-converting enzyme; ARBs: angiotensin receptor blockers; IQR: interquartile range.

### Effects of exercise training

Nineteen patients in the RG completed the protocol; two patients withdrew for personal reasons and one due to a cardiovascular event. Adherence to the exercise training program was 76.7 ± 19.0% of sessions.

The mean duration of AET was 39.3 ± 3.7 minutes. Mean HR during AET was 78.7 ± 5.4 bpm, corresponding to 50.1 ± 4.3% of the maximum age-related HR and 96.7 ± 11.3% of the VT1 HR. The angina threshold HR during AET was 81.3 ± 8.9 bpm, which was near close to the angina/ischemic threshold identified during CPET. Fifteen patients (73.7%) experienced angina during AET, and five of them (26.3%) required sublingual nitrate during at least one AET session. Mean systolic blood pressure and diastolic blood pressure during AET was 121.7 ± 14.1 mmHg and 70.8 ± 5.6 mmHg, respectively.

Despite the presence of angina, no arrhythmias or ST-segment depressions requiring cessation of exercise occurred during AET.^20^Regarding resistance training, patients did not report angina symptoms and/or any muscle discomfort; thus, resistance training was carried out as planned.

### Clinical evaluation

After 12 weeks on the protocol, no significant differences were observed between the groups in terms of CCS angina functional class, AAW, SANCW, or clinical and laboratory parameters ( Table 4 ).

**Table 4:** – Changes in clinical parameters in each group

Variable	GR		GC		Valor p		
	Pre	Post	Pre	Post	G	M	Int.
**Clinical data**							
Angina functional class CCS, n					0.81	0.01	0.26
0		1 (5.3)					
I		6 (31.6)		3 (14.3)			
II	7 (31.8)	6 (31.6)	10 (43.5)	9 (42.9)			
III	6 (27.3)	1 (5.3)	5 (21.7)	3 (14.3)			
IV	9 (40.9)	5 (26.3)	8 (34.8)	6 (28.6)			
Angina frequency, n	11.6±10.1	7.18±12.5	8.8±11.1	10.4±11.1	0.76	0.26	0.05
Nitrate consumption, n	4.6±6.5	4.5±6.9	4.7±7.7	5.8±9.1	0.76	0.51	0.55
SBP, mmHg	124±16.8	119±16.5	123±17.6	121±20.2	0.87	0.26	0.70
DBP, mmHg	76.3±9.4	72.3±9.6	74.9±12.0	72.5±12.7	0.83	0.10	0.68
HR, bpm	63.3±6.5	61.7±5.7	59.3±7.4	63.3±9.6	0.51	0.25	0.02
BMI, kg/m^2^	29.7±4.6	29.8±5.4	29.9±4.1	28.7±4.2	0.71	0.78	0.31
**Laboratory data**							
Fasting glucose, mg/dL	131±31.5	123±26.8	149±72.8	143±82.8	0.22	0.53	0.66
HBA1C, %	6.7±1.3	6.5±0.9	7.0±1.8	6.8±1.6	0.35	0.76	0.37
LDL-c, mg/dL	75.1±25.4	72.8±23.2	89.9±40.9	93.2±42.9	0.145	0.56	0.94
HDL-c, mg/dL	39.7±9.0	40.0±9.9	47.3±12.7	48.1±10.3	0.033	0.79	0.25
TG, mL/g	143±42.6	136±50	113±41	141±71.1	0.331	0.18	0.01
CRP, mg/dL	1.68±2.3	1.8±2.7	2.7±2.6	2.1±2.0	0.355	0.86	0.42

Data is shown in mean ± SD or n: number (%). RG: rehabilitation group; CG: control group; G: group; M: moment; Int.: interaction; Pre: before rehabilitation or medical treatment alone; Post: after rehabilitation or medical treatment alone; CCS: Canadian Cardiovascular Society; SBP: systolic blood pressure; DBP: diastolic blood pressure; HR: heart rate; BMI: body mass index; HBA1C: glycosylated hemoglobin; LDL: low density lipoprotein; HDL: high density lipoprotein; CRP: C-reactive protein. Data are expressed as mean ± standard deviation or number (%). G: RG vs CG. M: pre vs post. Int.: combined effect between group and moment.

### ESE evaluation

There was a significant improvement in peak load, angina quantification, exercise duration, and time to angina and ischemic thresholds between groups, as shown in Table 5 and Figure 2B, 2C, and 2D , respectively. No differences were found in mean LVEF, ischemic scores, peak HR, or HR at IT and AT ( Tables 5 and 6 ).

**Table 5:** – Changes in echocardiographic and cardiopulmonary parameters in each group

Variable	RG		CG		p value		
	Pre	Post	Pre	Post	G	M	Int.
**ESE**							
LVEF, %	54.5±7.6	53±7.7	50±9.9	48.8±9.9	0.06	0.03	0.67
Rest score, n	1.28±0.22	1.28±0.16	1.45±0.37	1.37±0.28	0.06	0.51	0.35
Exercise score, n	1.49±0.28	1.36±0.21	1.62±0.38	1.49±0.29	0.08	<0.01	0.62
Delta score, au	0.21±0.16	0.08±0.11	0.16±0.11	0.12±0.11	0.95	<0.01	0.10
Exercise Duration, s	291±89.5	344±143	277±110	266±93.7	0.07	0.02	<0.01
Peak load (W), J/s	34.7±17.8	46.2±23.8	35.2±24.2	35±21.4	0.31	<0.01	<0.01
Angina quantification, n	5.2±3.2	4.4±2.6	6.4±2.3	6.6±2.5	0.01	0.04	<0.01
Peak (HR), bpm	96.1±9.7	95.5±18	99.5±15.8	94.8±11.9	0.84	0.58	0.07
IT (HR), bpm	90.5±7.2	93.5±12.7	93.6±15.7	91.6±10.5	0.93	0.88	0.20
IT time, s	241±86.4	299±136	210±74.9	214±83.4	0.02	<0.01	<0.01
AT (HR), bpm	89.1±7.5	89.2±8.9	92.1±16.5	90.6±10.2	0.60	0.70	0.65
AT (time), s	188±80.6	265±160	204±95.2	161±69.8	0.09	0.12	<0.01
**CPET**							
VO_2 peak_, mL.kg.min^-1^	15.9±3.3	16.3±4.3	15.7±3.6	15.6±3.5	0.51	0.72	0.46
VO_2_predicted, %	63.8±19.2	64.5±18.3	60.2±14.8	61.3±10.3	0.31	0.80	0.78
Angina quantification, n	6.4±1.5	6.5±1.8	6.2±2.0	6.4±2.7	0.95	0.85	0.66
VT1 (HR), bpm	82.1±9.6	82.7±9.7	87.6±16.0	83.6±10.5	0.34	0.45	0.37
AT (time), s	198±66	322±174	248±132	287±94	0.86	<0.05	0.07
AT (HR), bpm	85.9±13.9	88±13.1	92.5±16.3	90.7±15.2	0.33	0.95	0.36
RQ, au	0.9±0.99	1.0±0.1	1.0±0.1	1.0±0.1	0.93	0.74	0.01
Pulse oxygen, mL/beat	13.6±3.7	14.7±3.4	12:3±3:1	12.9±3.6	0.18	<0.01	0.43
Covered distance, m	312±151	443±205	360±155	363±130	0.76	<0.01	<0.01
Total duration, s	345±124	435±181	327±141	371±128	0.18	<0.01	<0.01

Data is shown in mean ± SD . RG: rehabilitation group; CG: control group; G: group; M: moment; Int.: interaction; Pre: before rehabilitation or medical treatment alone; Post: after rehabilitation or medical treatment alone; ESE: exercise stress echocardiography; LVEF: left ventricular ejection fraction; HR: heart rate; IT: ischemic threshold; AT: angina threshold; CPET: cardiopulmonary exercise testing; VT1: ventilatory threshold; RQ: respiratory quotient. G: RG vs CG. M: pre vs post. Int.: combined effect between group and moment.

**Figure 2:** – Change in exercise duration in cardiopulmonary exercise test, exercise duration, time to ischemic threshold and time to angina threshold in exercise stress echocardiography by study group.

**Table 6:** – Effect Sizes (Cohen’s d) and their Confidence Intervals (CI)

Variables (RGpost vs. CGpost)	Cohen’s d	CI 95% Lower		CI 95% Upper	G	M	Int.
ESE							
Exercise Duration, s	0.65	-5.79	186.32		0.07	0.02	<0.01
Peak load (W), J/s	0.49	-7.18	31.76		0.31	<0.01	<0.01
Angina quantification, n	0.87	-5.58	-0.63		0.01	0.04	<0.01
IT time, s	0.80	5.79	191.09		0.02	<0.01	<0.01
AT (time), s	0.96	0.27	223.25		0.09	0.12	<0.01
CPET							
Covered Distance, m	0.47	-66.45	225.76		0.76	<0.01	<0.01
Total Duration, s	0.41	-32.31	231.41		0.18	<0.01	<0.01

RGpost: rehabilitation group after rehabilitation period; CG: control group after medical treatment alone; G: group; M: moment; Int.: interaction; ESE: exercise stress echocardiography; IT: ischemic threshold; AT: angina threshold; CPET: cardiopulmonary exercise testing; G: RG vs CG. M: pre vs post. Int.: combined effect between group and moment.

### CPET performance evaluation

There was a significant increase in CPET exercise duration and distance covered in RG, as demonstrated in Table 5 and Figure 2A . No significant differences were observed in VO_2_peak or other CPET variables ( Tables 5 and 6 ).

## Discussion

For the first time, the safety and efficacy of ECR in improving symptoms, ischemic burden, and functional capacity were objectively evaluated in patients with RA. Our results demonstrate that a 12-week ECR program, performed near the AT and/or VT1, was safe and yielded positive effects on exercise duration, angina intensity, and ESE ischemic and angina thresholds.

Since RA was first documented as a clinical condition in the early 2000s, ECR has been considered a treatment option. However, strong evidence supporting its safety and guidance on its application in the RA context has remained lacking.^6 , 14 , 15 , 26 , 27^

In our study, patients experienced debilitating angina due to myocardial ischemia despite using at least three classes of antianginal agents and were not eligible for coronary intervention due to the complexity of coronary obstructive disease. Consequently, these patients presented a had a high-risk profile for exercise-induced adverse cardiac events,^8^and their physical limitations made both the prescription and safe execution of exercise particularly challenging – especially in achieving the target of moderate-intensity exercise.

However, exercising training guided by CPET parameters and angina threshold – allowing the experience of angina symptoms up to level 3 on a 10-point scale – did not increase the risk of cardiovascular events during the study period. These data are supported by our previous data, which demonstrated that an acute aerobic exercise session does not alter hs-cTnT in patients with RA, suggesting that no significant myocardial injury occurred when exercise was performed according to our prescribed protocol.^15^

Despite the lack of improvement in clinical parameters defined by angina functional class (CCS), AAW, and SANCW – self-reported variables – we consider the improvement in exercise test parameters a key indicator of ECR’s positive effect. The increase in exercise duration, reduction in angina intensity, and delayed onset of angina can positively impact quality of life, which is the central focus of RA management. Moreover, exercise test parameters are objective measures, making them reliable indicators of actual improvement in RA patients in clinical practice.

No significant improvement was observed in VO_2_peak. Due to physical limitations stemming from a low angina threshold, maintaining the target moderate-intensity AET was challenging – and at times unfeasible – which may explain the lack of improvement in VO_2_peak. Nevertheless, even without an increase in VO_2_peak (the most important prognostic parameter in CPET), the increase in distance covered during exercise testing can be interpreted as a clinical improvement, as it is also considered a prognostic marker in patients with heart disease.^28^

The improvement in exercise performance (and the non-concomitant change in VO_2_peak) may be partially explained by early adaptations, such as neurological and muscular changes induced by resistance training.^10 , 29 , 30^

Therefore, RC is a feasible adjuvant therapy for patients with RA, supporting its clinical indication. We believe that 12-week ECR is a starting point to exercise benefits in RA patients and sustained ECR may promote additional positive effects and improve quality of life.

### Limitations

The results of this analysis must be interpreted within the context of potential limitations.

The sample size calculation was based on the only previous study that evaluated the effect of cardiac rehabilitation in patients with RA, despite different pre-determined outcomes.

The study presented difficulties of inclusion due to strict inclusion criteria.

## Conclusion

A 12-week ECR was safe and effective in improving functional capacity and ESE ischemic burden in patients with RA.
